# Density of states in the presence of spin-dependent scattering in SF bilayers: a numerical and analytical approach

**DOI:** 10.3762/bjnano.13.117

**Published:** 2022-12-01

**Authors:** Tairzhan Karabassov, Valeriia D Pashkovskaia, Nikita A Parkhomenko, Anastasia V Guravova, Elena A Kazakova, Boris G Lvov, Alexander A Golubov, Andrey S Vasenko

**Affiliations:** 1 HSE University, 101000 Moscow, Russia; 2 Sechenov First Moscow State Medical University, 119991 Moscow, Russia; 3 Faculty of Science and Technology and MESA Institute for Nanotechnology, University of Twente, 7500 AE Enschede, Netherlands; 4 Moscow Institute of Physics and Technology, 141700 Dolgoprudny, Russia; 5 I. E. Tamm Department of Theoretical Physics, P. N. Lebedev Physical Institute, Russian Academy of Sciences, 119991 Moscow, Russia

**Keywords:** density of states, Josephson junctions, proximity effect, superconductivity, superconductor/ferromagnet hybrid nanostructures

## Abstract

We present a quantitative study of the density of states (DOS) in SF bilayers (where S is a bulk superconductor and F is a ferromagnetic metal) in the diffusive limit. We solve the quasiclassical Usadel equations in the structure considering the presence of magnetic and spin–orbit scattering. For practical reasons, we propose the analytical solution for the density of states in SF bilayers in the case of a thin ferromagnet and low transparency of the SF interface. This solution is confirmed by numerical calculations using a self-consistent two-step iterative method. The behavior of DOS dependencies on magnetic and spin–orbit scattering times is discussed.

## Introduction

It is well-known that superconductivity can be induced in a non-superconducting metal in hybrid structures due to the proximity effect [1– 7]. For instance, in NS bilayers (where N denotes a normal metal and S denotes a superconductor), the superconducting correlations penetrate into the normal metal layer over a characteristic decay length ξ*_n_* = 
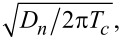
 where *D* *_n_* is the diffusion constant in the normal metal and *T* *_c_* is the transition temperature. When a superconductor S is combined with a ferromagnetic layer F, forming an SF bilayer, the superconductivity leaks into the ferromagnetic region over the characteristic length ξ*_h_* = 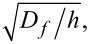 where *D* *_f_* is the diffusion constant in F layer and *h* is the exchange field in the ferromagnetic layer [1]. Not only superconductivity is substantially suppressed due to the exchange field, but also Cooper pairs gain a finite center of mass momentum, which leads to the oscillatory behavior of the Cooper pair wave function. These oscillations can be described in the diffusive limit in the framework of the so-called Usadel equations, which are written in terms of the quasiclassical Green’s functions. This approach proved to be very powerful for the description of the proximity effect in diffusive superconducting hybrids [1– 3 8– 11].

The scientific community has been examining the proximity effect in SF hybrid structures already for a long while. It has been found that the oscillatory behavior of the superconducting wave function can lead to various interesting phenomena that can be observed experimentally [1– 2]. For instance, the superconducting transition temperature shows non-monotonous and, in some cases, oscillatory behavior in multilayered SF structures [12– 17]. Recently, it has been shown theoretically that similar behavior can be observed in S/TI structures with non-uniform magnetization patterns on the surface of a 3D topological insulator (TI) [18]. The Josephson critical current demonstrates damped oscillatory behavior as a function of the thickness of the ferromagnetic layer in SFS Josephson junctions [19– 47]. Similarly, the density of states (DOS) also demonstrates a damped oscillatory dependence as a function of the F layer thickness in SF systems [48– 52].

The density of states is one of the crucial spectral characteristics of the proximity effect in superconducting hybrid structures. For example, the DOS calculation is essential for the quasiparticle current computation in SIFS (where I denotes an insulating layer) [29, 51, 53– 57] or SFIFS tunneling Josephson junctions [58]. Therefore, computation of the DOS is also needed for many actively studied areas of research, including the thermospin [59– 60] and thermoelectric [61– 66] effects, spin and heat valves [67– 75], as well as nanoscale refrigerators [76– 78]. Presently, the DOS structure at the free edge of a normal metal layer in NS bilayers is well-known [1– 3 79]. It has a so-called mini-gap at the subgap energies *E <* Δ (where Δ is the superconducting gap), whose magnitude depends on the NS interface parameters and the thickness of the N layer [79– 80]. Replacing the N layer with a ferromagnetic metal F results in a more sophisticated DOS structure since there is a non-zero exchange field, which causes spin-split densities of states for two spin populations of electrons [1– 3 81]. More general considerations should also include possible spin-flips as well as spin–orbit scattering processes in the ferromagnetic region [82].

In this work, we consider a diffusive SF bilayer, assuming a relatively low interface transparency and the presence of magnetic and spin–orbit scattering. For this purpose, the Kupriyanov–Lukichev (KL) boundary conditions at the superconductor/ferromagnet interface are perfectly suitable [83]. We build the model employing the Usadel equations. The quasiclassical Usadel equations are based on the Matsubara Green’s functions and represent a powerful microscopic tool for the treatment of the diffusive superconducting hybrid structures. This approach is valid as long as the elastic scattering length *l* is much smaller than the superconducting coherence length ξ = 
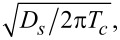
 where *D* *_s_* is the diffusion constant in the superconductor. Previously, the DOS in SF bilayers has been studied numerically [84– 85]. We revisit this question and propose an analytical model to describe the influence of spin-flip and spin–orbit scattering on the DOS behavior. Then, we provide a comparison with the exact numerical calculation using a self-consistent two-step iterative method. Furthermore, we briefly discuss the consequences of the different kinds of scattering on the current–voltage characteristics in SFIFS junctions. We do not consider any additional effects in the SF boundary such as spin-dependent interfacial phase shifts (SDIPS). The effect of SDIPS on the DOS behavior in SFIFS junctions has been studied both analytically [86] and numerically [87].

The paper is organized as follows. In the section (“Model”) we formulate the theoretical model. In the following sections, the derivation of the analytical results is presented. We discuss the calculations in the section (“Results and Discussion”), and finally we summarize the results in the last section (“Conclusion”).

## Model

**Figure 1:**
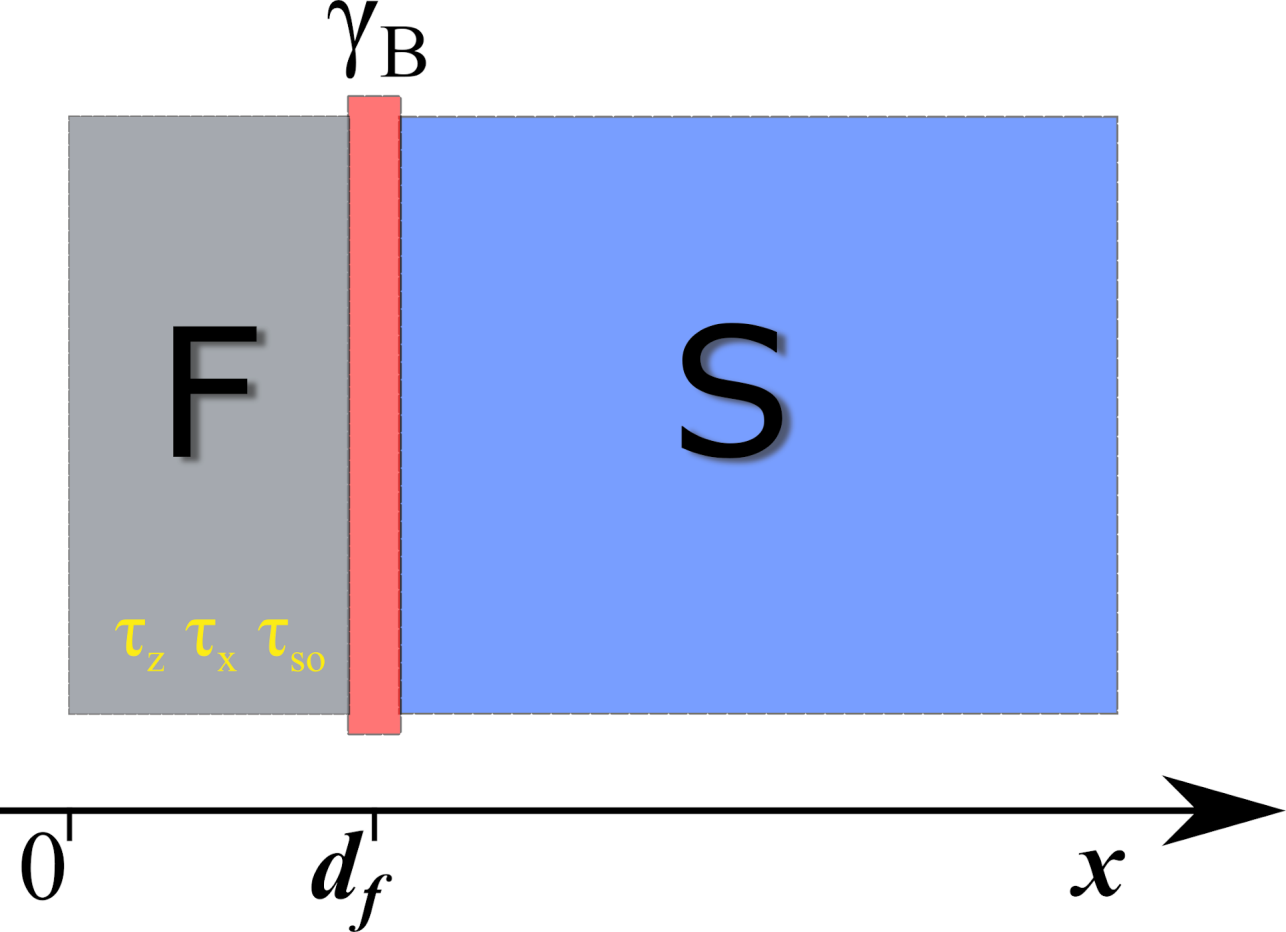
Geometry of the SF bilayer. We consider the SF interface to be a tunnel barrier. Here, γ*_B_* is the interface transparency parameter.

The theoretical model of the SF structure under consideration is depicted in Figure 1. It consists of a ferromagnetic layer with thickness *d* *_f_* and a superconducting electrode along the *x* direction. The SF interface is characterized by the dimensionless parameter γ*_B_* =*R* *_B_*σ*_n_*/ξ*_f_*, where *R* *_B_* is the resistance of the SF interface in units Ω·m^2^, σ*_n_* is the conductivity of the F layer [88–89], ξ*_f_* =
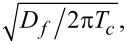
 *D* *_f_* is the diffusion coefficient in the ferromagnetic metal, and *T* *_c_* is the critical temperature of the superconductor [1–2]. We assume ℏ = *k*_B_ = 1. We also assume that the SF interface is not magnetically active. We will consider the diffusive limit in this model and neglect the nonequilibrium effects in the structure [90–92].

Our goal is to find the DOS of a single SF bilayer, which can be done by solving the Usadel equations in the ferromagnetic and superconducting layers. We employ the θ parametrization of the normal and anomalous quasiclassical Green’s functions, *G* = cosθ and *F* = sinθ, respectively. In the spin space, the anomalous functions are parameterized as


[1] $$\hat{F}=\left(\begin{array}{cc} 0 & \sin\theta_{↑}\\ -\sin\theta_{↓} & 0 \end{array}\right),$$

and we can write the Usadel equations in the F layer as [4,51,82,84,93]


[2] $$\begin{array}{c} \frac{D_{f}}{2}\frac{\partial^{2}\theta_{f↑\left(↓\right)}}{\partial x^{2}}=\left(\omega \pm ih+\frac{1}{\tau_{z}}\cos\theta_{f↑\left(↓\right)}\right)\sin\theta_{f↑\left(↓\right)}\\ +\frac{1}{\tau_{x}}\sin\left(\theta_{f↑}+\theta_{f↓}\right)\pm \frac{1}{\tau_{so}}\sin\left(\theta_{f↑}-\theta_{f↓}\right), \end{array}$$

where the positive and negative signs correspond to the spin-up (↑) and spin-down (↓) states, respectively. In terms of the electron fermionic operators ψ, the spin-up state corresponds to the anomalous Green’s function *F*_↑_ ∼ ⟨ψ_↑_ψ_↓_⟩, while the spin-down state corresponds to *F*_↓_ ∼ ⟨ψ_↓_ψ_↑_⟩. We use the Matsubara Green’s functions, hence, ω = 2 π*T*(*n* + 1/2) are the Matsubara frequencies [94]. The exchange field of the ferromagnet is *h*, and the scattering times are labeled here as τ*_z_*, τ*_x_* and τ*_so_*. The parameter τ*_z(x)_* corresponds to the magnetic scattering parallel (perpendicular) to the quantization axis, and τ*_so_* is the spin–orbit scattering time [82,84,95].

In the model under consideration, we assume a rotational symmetry around the 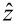 axis, that is, τ*_x_* = τ*_y_*. We consider the possibility of anisotropic magnetic scattering times. These can occur in ferromagnetic superconductors where magnetic disorder can be characterized by two scattering times τ*_x_* = τ*_y_* and τ*_z_* [82]. The Usadel equations employed in this work are the special case of the more general formalism derived by Ivanov and co-workers [96]. Thus, the equations that we use in our calculations are exactly a quasiclassical limit of the model derived in [96] except that in our model we also include spin–orbit scattering. Ivanov et al. describe the spin-flip scattering via a symmetric matrix of scattering rates 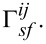 Depending on the symmetry of the problem, the number of parameters can be different (up to six), for example, in the most symmetric isotropic case, the spin-flip matrix is diagonal and described by one scattering time (τ*_x_* = τ*_y_* = τ*_z_*). In the case of a ferromagnetic ordering in which disorder is anisotropic (one axis is special), the spin-flip rates in the directions along the ferromagnetic axis and perpendicular to it may be different. In this case, as we mentioned earlier, two scattering times would be necessary.

In the S layer, the Usadel equation has the following form [93]:


[3] $$\frac{D_{s}}{2}\frac{\partial^{2}\theta_{s}}{\partial x^{2}}=\omega \sin\theta_{s}-\Delta \left(x\right)\cos\theta_{s}.$$

Here, *D* *_s_* is the diffusion coefficient in the superconductor, and Δ(*x*) is the superconducting order parameter (pair potential). From the Usadel equations, it can be shown that there is a symmetry relation between θ_↑_ and θ_↓_: θ_↑_(*E*) =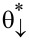 (−*E*), where *E* is the energy (ω*_n_* → −*iE*) and ^*^ is the complex conjugation. Equation 2 and Equation 3 should be supplemented with the self-consistency equation for the coordinate dependence of superconducting order parameter Δ,


[4] $$\Delta \left(x\right)\ln\frac{T_{c}}{T}=\pi T\sum_{\omega >0}\left(\frac{2\Delta \left(x\right)}{\omega }-\sin\theta_{s↑}-\sin\theta_{s↓}\right).$$

The resulting system must be complemented by the boundary conditions at the outer boundary of a ferromagnet,


[5] $${\left(\frac{\partial \theta_{f}}{\partial x}\right)}_{x=0}=0,$$

and the Kupriyanov–Lukichev boundary conditions at the FS interface [83],


[6] $$\xi_{f}\gamma {\left(\frac{\partial \theta_{f}}{\partial x}\right)}_{x=d_{f}}=\xi_{s}{\left(\frac{\partial \theta_{s}}{\partial x}\right)}_{x=d_{f}},$$

[7] $$\xi_{f}\gamma_{B}{\left(\frac{\partial \theta_{f}}{\partial x}\right)}_{x=d_{f}}=\sin{\left(\theta_{s}-\theta_{f}\right)}_{x=d_{f}}.$$

Here γ = ξ*_s_*σ*_n_*/ξ*_f_*σ*_s_*, σ*_s_* is the conductivity of the S layer, and ξ*_s_* =
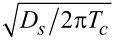
 is the superconducting coherence length. The indices of the θ-parameterized Green’s functions are omitted, since there is no mixing between the components θ_↑_ and θ_↓_. In other words, we are not considering spin-active interfaces. In the case of spin-active barriers, one should use the boundary conditions introduced in [87,97–98], rather than the standard Kupriyanov–Lukichev boundary conditions in Equation 6 and Equation 7. The parameter γ determines the strength of superconductivity suppression in the S layer by the ferromagnet F (inverse proximity effect). For instance, when γ ≫ 1, the inverse proximity effect is very strong, and the order parameter is heavily suppressed near the SF interface compared to its bulk value. On the contrary, when γ = 0, there is no suppression of the order parameter because there is no inverse proximity effect. In all numerical simulations, we assume that γ ≪ 1, that is, there is almost no superconductivity suppression in the superconductor. The transparency parameter γ*_B_* is proportional to the interface resistance. In the regime of a fully transparent junction, γ*_B_* = 0, the proximity effect is the strongest, and the θ functions are continuous at the SF interface while γ*_B_* ≫ 1 corresponds to the tunnel junction limit, that is, there is no mutual impact between superconducting layer S and ferromagnetic layer F.

To complete the boundary problem, we also set a boundary condition at *x* = +∞:


[8] $$\theta_{s}\left(+\infty \right)=\arctan\left(\frac{\Delta }{\omega }\right),$$

where the Green’s functions take the well-known bulk BCS form. The Green’s function method allows us to compute the DOS at the outer F boundary by solving the resulting system of equations above.

The DOS at the outer F boundary *N* *_f_*(*E*) is normalized to the DOS in the normal state and can be written as


[9] $$N_{f}\left(E\right)=\left[N_{f↑}\left(E\right)+N_{f↓}\left(E\right)\right]/2,$$

where *N* *_f↑_*_(↓)_(*E*) are the spin-resolved DOS written in terms of the spectral angle θ,


[10] Nf↑(↓)(E)=Re[cosθf↑(↓)(iω→E+i0)].

To calculate Equation 10, we use a self-consistent two-step iterative method. In the first step, we calculate the pair potential coordinate dependence Δ(*x*) using the self-consistency equation (Equation 4) in the S layer. Then, by proceeding to the analytical continuation in Equation 2 and Equation 3 over the quasiparticle energy *i*ω →*E* +*i*0 and using the Δ(*x*) dependence obtained in the previous step, we find the Green’s functions by repeating the iterations until convergency is reached.

### The DOS in the limit of small F layer thickness

In this section, we obtain the analytical result assuming *d* *_f_* ≪ min(ξ*_f_*,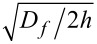 ), which is the case in a thin and weak ferromagnet. Under the condition γ = 0, we can neglect the suppression of superconductivity in the superconductor. Hence, the problem can be reduced to the rigid boundary condition when the order parameter in the S layer is set to its bulk value Δ_0_. We will keep all scattering terms in the solution to obtain a more general result. In this case, we can expand the solution of the Usadel equations up to the second order in small spatial gradients. The θ*_f_* functions can be approximated in the following way:


[11] $$\theta_{f↑}=A_{↑}+B_{↑}x+C_{↓}x^{2},$$

[12] $$\theta_{f↓}=A_{↓}+B_{↓}x+C_{↓}x^{2},$$

where the coefficients *A* *_↑_*_(↓)_,*B* *_↑_*_(↓)_,*C* *_↑_*_(↓)_ are determined from the boundary conditions.

Inserting the solution in Equation 11 into the Usadel equation in the F layer (Equation 2), we get,


[13] $$\begin{array}{c} C_{↑\left(↓\right)}=\frac{1}{2}\left[\left(\omega_{n}\pm ih\right)\sin A_{↑\left(↓\right)}+\frac{1}{2}\alpha_{z}\sin2A_{↑\left(↓\right)}\right]\\ +\frac{1}{2}\left[\alpha_{x}\sin\left(A_{↑}+A_{↓}\right)\pm \alpha_{so}\sin\left(A_{↑}-A_{↓}\right)\right]. \end{array}$$

For convenience, we introduced the scattering rate parameters α*_z_* = 1/τ*_z_*Δ, α*_x_* = 1/τ*_x_*Δ, and α*_so_* = 1/τ*_so_*Δ. To find the coefficients, we utilize the boundary conditions in Equation 5 and Equation 7,


[14] $${\left(B_{↑\left(↓\right)}+2C_{↑\left(↓\right)}x\right)}_{x=0}=0,$$

[15] $$\xi_{f}\gamma_{B}{\left(B_{↑\left(↓\right)}+2C_{↑\left(↓\right)}x\right)}_{x=d_{f}}={\sin\left(\theta_{s}-A_{↑\left(↓\right)}\right)|}_{x=d_{f}}.$$

From the first equation we obtain *B*_↑(↓)_ = 0, while the second equation results in the expression for *A* *_↑_*_(↓)_.

Now, we will discuss a ferromagnet with strong uniaxial anisotropy, in which case the perpendicular fluctuations of the exchange field are suppressed (α*_x_* ∼ 0). For simplicity, we also assume the ferromagnet with weak spin–orbit interactions and also neglect the spin–orbit scattering time α*_so_*. Finally, assuming α*_x_* = α*_so_* = 0 and keeping the solution to the lowest order, the equation for θ*_f_* takes the form


[16] $$\begin{array}{l} \gamma_{B}d_{f}\left(\omega_{n}\pm ih\right)\tan\theta_{f↑\left(↓\right)}\\ +\cos\theta_{s}\tan\theta_{f↑\left(↓\right)}+\alpha_{z}\gamma_{B}d_{f}\sin\theta_{f↑\left(↓\right)}=\sin\theta_{s}, \end{array}$$

where sinθ*_s_* = Δ_0_ /
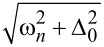
 and cosθ*_s_* = ω*_n_*/
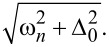
 Here, Δ_0_ is the bulk value of the pair potential. The equation above can be used for further semi-analytical calculations of the DOS for the case of a thin F layer with the magnetic scattering rate α*_z_*. When α*_z_* = 0, Equation 16 reduces to the well-known result (see, for example, [81] or [86]),


[17] $$\tan\theta_{f↑\left(↓\right)}=\frac{\sin\theta_{s}}{\left(\omega_{n}\pm ih\right)\gamma_{B}d_{f}+\cos\theta_{s}}.$$

### Analytical solution in the low proximity limit and small F layer thickness

In this section, we perform further analytical calculations of the anomalous Green’s function in the F layer based on the results of the previous section. We then analyze the effect of various scattering rates on the superconducting correlations, including the odd-frequency triplet component, which is generated in the adjacent ferromagnet.

The expression for θ-parameterized Green’s functions can be found from the boundary conditions (Equation 14),


[18] $$\begin{array}{c} \gamma_{B}d_{f}\left[\left(\omega_{n}\pm ih\right)+\alpha_{z}\cos\theta_{f↑\left(↓\right)}\right]\sin\theta_{f↑\left(↓\right)}\\ +\gamma_{B}d_{f}\left[\alpha_{x}\sin\left(\theta_{f↑}+\theta_{f↓}\right)\pm \alpha_{so}\sin\left(\theta_{f↑}-\theta_{f↓}\right)\right]\\ =\sin\left(\theta_{s}-\theta_{f↑\left(↓\right)}\right). \end{array}$$

In order to simplify the calculation and the final form of the solution θ*_f_*, we consider only positive Matsubara frequencies ω*_n_* and perform further linearization of Equation 18, which is justified in the low-proximity limit. Then, we obtain


[19] $$\begin{array}{l} \theta_{f↑\left(↓\right)}=\\ \frac{\sin\theta_{s}\left(\cos\theta_{s}+\gamma_{B}d_{f}\left(\alpha_{z}+2\alpha_{so}+\omega_{n}\mp ih\right)\right)}{\gamma_{B}^{2}d_{f}^{2}\left[h^{2}-{\left(\alpha_{x}-\alpha_{so}\right)}^{2}\right]+{\left[\cos\theta_{s}+\gamma_{B}d_{f}\left(\sum \alpha_{i}+\omega_{n}\right)\right]}^{2}}. \end{array}$$

Here, Σα*_i_* denotes the sum of all the scattering rates. The above solution is true for thin ferromagnetic layers in the low-proximity limit. A more general analytical solution can be obtained for arbitrary thicknesses using the linearized Usadel equations. In order to find the DOS in the proposed limit, we expand the θ-parametrized normal Green’s function around small values of θ*_f_*. In this case, we have


[20] Nf↑(↓)(E)≈1−12Re[θf↑(↓)2(ωn→−iE)],

and, to calculate the total DOS, we need to sum the contributions from two spin populations using Equation 9.

## Results and Discussion

In the present section we outline the main results, including both numerical and analytical calculations. The following parameters are fixed throughout the section: *T* = 0.1*T* *_c_*, γ = 0.05, and *d* *_f_* = 0.5ξ*_f_* *.* First, we discuss general features of the DOS in an SF bilayer in the absence of any scattering. Then the effect of the spin-dependent scattering on the key DOS features in two relevant cases is discussed (see below) and finally, we present the analytical result and compare it with the numerically calculated DOS.

### Evolution of the DOS in SF bilayer

It is instructive to discuss the key features of the DOS in an SF bilayer first. That is why, in this section, we briefly discuss the evolution of the DOS for different values of the exchange field *h* and the barrier transparency γ*_B_*. All the scattering is assumed to be absent for simplicity α*_m_* = α*_x_* = α*_so_* = 0 in this subsection.

**Figure 2:**
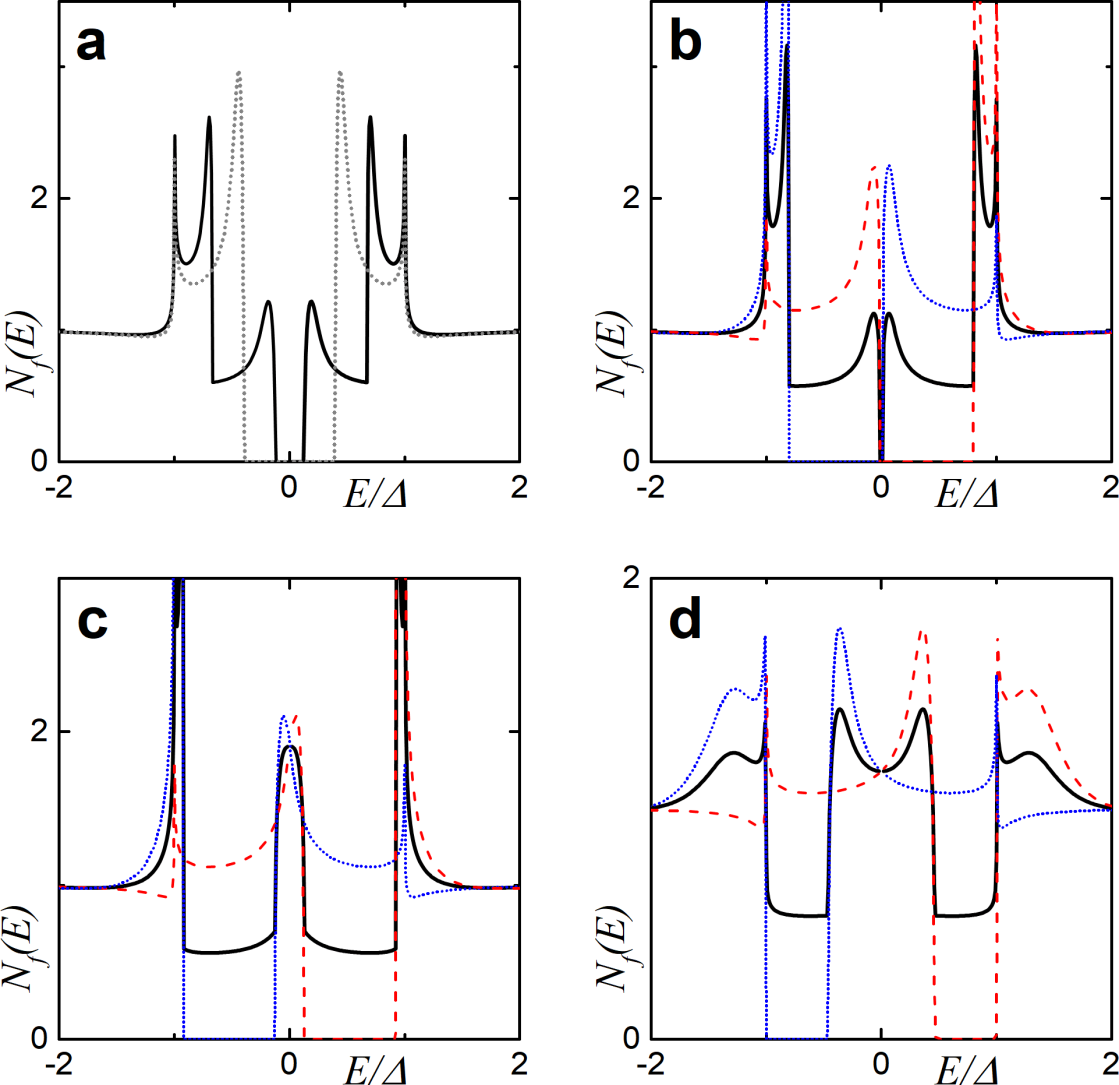
The evolution of the DOS plotted for increasing values of the exchange field *h*. Here, γ*_B_* = 5, *d* *_f_* = 0.5ξ*_f_*. In plot (a), the gray dotted line represents the case of *h* = 0 (i.e., the NS bilayer). In plots (a)–(d), black solid lines correspond to the total DOS while red dashed lines show *N* *_f↑_* (*E*) and blue dash-dotted lines show *N* *_f↓_*(*E*). (a) Black solid line calculated for *h* = 0.4Δ, and all lines are calculated for (b) *h* = 0.8Δ, (c) *h* = Δ, and (d) *h* = 1.6Δ.

In Figure 2, we observe the influence of an increasing exchange field *h* on the DOS structure calculated for γ*_B_* = 5. In particular, we can see the evolution of the DOS peaks. For *h* = 0, that is, for the case of an SN bilayer, we see the well-known DOS structure with the characteristic mini-gap at energies *E <* Δ (Figure 2a, black dotted line) [79]. This proximity-induced mini-gap originates from the effective backscattering of the quasiparticles at the SN interface due to a finite interface resistance [99]. As *h* increases, the DOS splits for the spin-up and spin-down electrons, which results in the mini-gap peak splitting. For a certain value of *h*, the mini-gap closes, resulting in the DOS enhancement at zero energy as seen from Figure 2b and Figure 2c. This feature known as a zero-energy peak (ZEP) has been investigated both theoretically [100–103] and experimentally [49]. Another interesting peculiarity of the DOS is the appearance of the characteristic peak at *E* =*h*, which arises as the exchange field exceeds the superconducting gap *h >* Δ. Apparently, this peak arises from the evolution of the second spin-split peak due to a non-zero exchange field. The existence of such an effect offers a method of determining relatively small exchange field values in the F layer via DOS measurements [48,51,104].

**Figure 3:**
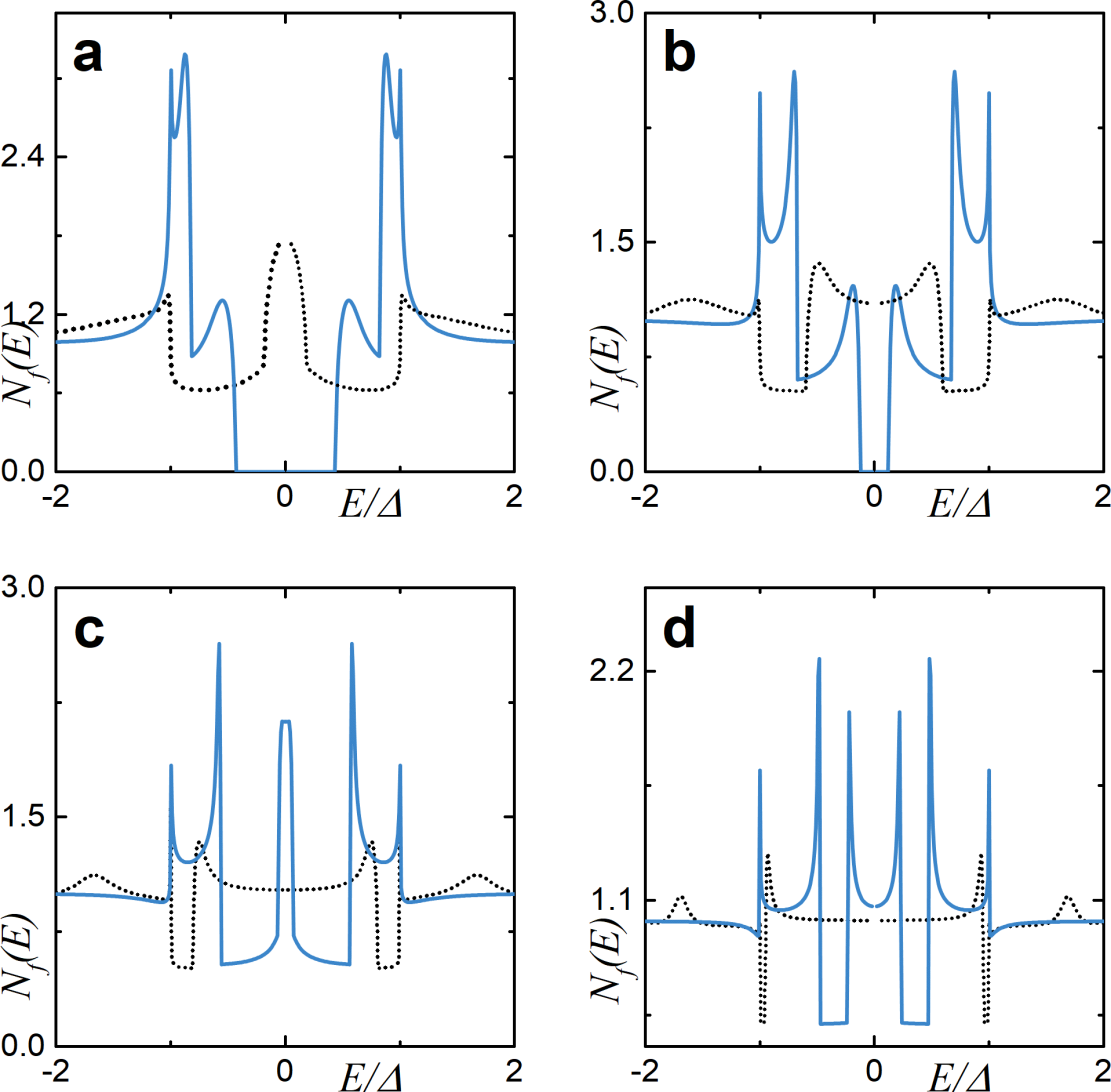
The evolution of the DOS plotted for increasing values of the SF interface transparency γ*_B_*. Here, *d* *_f_* = 0.5ξ*_f_*, the exchange field is *h* = 0.4Δ (blue solid line), and *h* = 1.7Δ (black dotted line). (a) γ*_B_* = 2, (b) γ*_B_* = 5, (c) γ*_B_* = 10, and (d) γ*_B_* = 25.

In Figure 3, the DOS evolution at increasing interface parameter γ*_B_* is shown. The blue solid line corresponds to the subgap exchange field *h* = 0.4Δ, whereas the black dotted line corresponds to *h* = 1.5Δ. From the figure we can notice that an increase of γ*_B_* also has a strong influence on the DOS structure. Sufficiently large interface resistance values can close the mini-gap and lead to the emergence of the ZEP (Figure 3c, blue solid line). However, the peak structure is different for the two exchange fields *h* as seen from the figure.

One can notice that the peak at *E* =*h* in Figure 3a is almost absent. The reason that we observe such a behavior is because we consider relatively small values of F layer thickness *d* *_f_* and transparency parameter γ*_B_*. More detailed analysis can be made in the limiting case of *d* *_f_* ≫ ξ*_f_* and γ*_B_* ≫ 1 and γ = 0 [51]. In the absence of any scattering, the analytical DOS expression for *E* ≥ Δ can be written in the following way:


[21] $$N_{f}\left(E\right)=1+\sum_{\pm }\frac{16\Delta^{2}\cos\left(\frac{2d_{f}}{\xi_{f}}\sqrt{\frac{\left|E\pm h\right|}{h}}\right)}{\left(E+\varepsilon \right){\left(\sqrt{E+\varepsilon }+\sqrt{2\varepsilon }\right)}^{2}}e^{-\frac{2d_{f}}{\xi_{f}}\sqrt{\frac{\left|E\pm h\right|}{h}}},$$

where ε = 
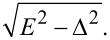
 One can clearly see the exponential asymptotic of the peak at *E* =*h* from Equation 21. We should not forget that Equation 21 is strictly valid only for large *d* *_f_*/ξ*_f_*, but nevertheless it may qualitatively explain why we do not see the peak at *E* =*h* for small a ratio of *d* *_f_*/ξ*_f_*. If this factor is relatively small, the variation of the exponent {−2(*d* *_f_*/ξ*_f_*)
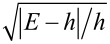
 } near the point *E* =*h* is also small. The peak is observable only for *h* of the order of a few Δ. For larger exchange fields, the peak is very difficult to observe since the energy-dependent prefactor of the exponent in Equation 21 decays as *E* ^−2^ for *E* ≫ Δ.

In what follows, we will examine the effect produced by both spin-flip and spin–orbit scattering on the DOS features, mostly focusing on the mini-gap and the DOS peak at *E* =*h*. Unlike previous results on this topic [84–85], we provide both numerical and analytical results for the DOS calculation. Although the analytical expressions have a rather narrow range of applicability such limiting cases are relevant for experiments.

### Effect of scattering on the DOS features

Now we discuss the influence of the finite scattering rates on the DOS features mentioned in the previous section. In this paper we consider two cases of the junction transparency: (i) intermediate interface transparency (γ*_B_* ≥ 1) and (ii) low interface transparency (γ*_B_* ≫ 1). In both cases, we fix the thickness of the F layer to *d* *_f_* = 0.5ξ*_f_*. Focusing on these cases allows us to discuss all major effects on the DOS features utilizing not only numerical solutions of the problem but also some analytical results, which will be presented below.

#### Intermediate interface transparency (γ*_B_* = 5)

**Figure 4:**
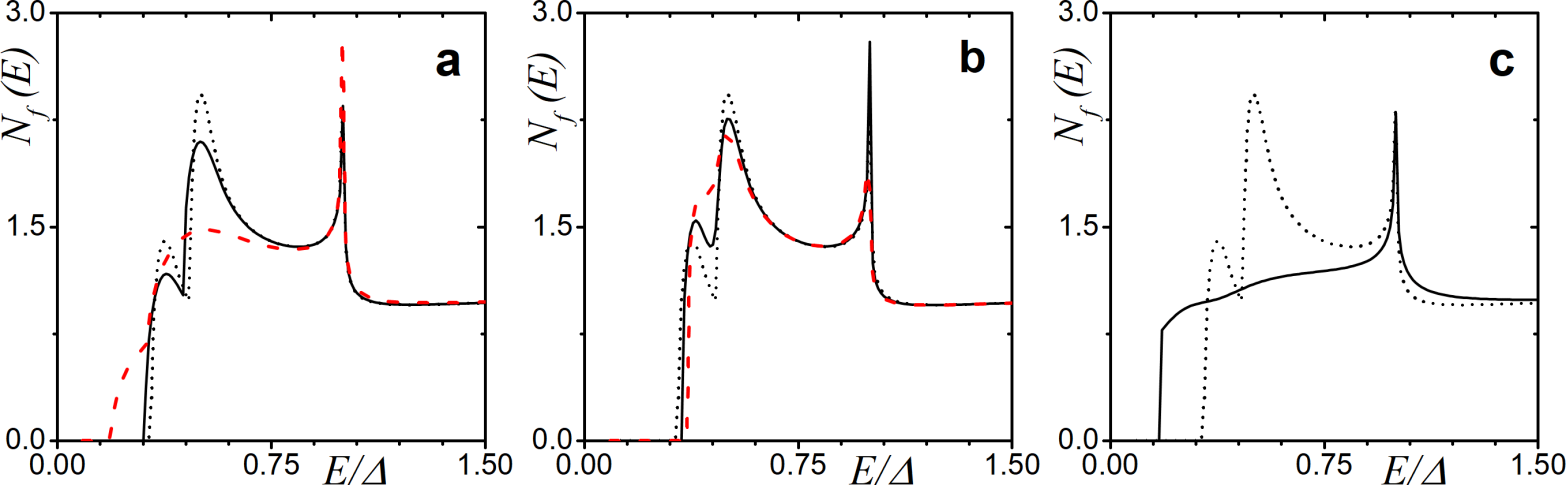
The DOS *N* *_f_*(*E*) at the free boundary of the F layer in the SF bilayer in the presence of magnetic and spin–orbit scattering, calculated numerically for different scattering times. The plots correspond to intermediate interface transparency γ*_B_* = 5, *h* = 0.1Δ, and *d* *_f_* = 0.5ξ*_f_*. Plot (a) corresponds to α*_z_* ≠ 0: α*_z_* = 0.01 (black solid line) and α*_z_* = 0.1 (red dashed line). Plot (b) corresponds to α*_so_* ≠ 0: α*_so_* = 0.05 (black solid line) and α*_so_* = 0.13 (red dashed line). Plot (c) corresponds to α*_x_* ≠ 0: α*_x_* = 0.2 (black solid line). The black dotted line represents *N* *_f_*(*E*) in the absence of any scattering.

Figure 4 depicts the DOS dependencies in the case of relatively low interface transparency (γ*_B_* = 5) in the presence of spin-flip and spin–orbit scattering. It is clearly seen that the decrease of the parallel magnetic scattering time leads to a smearing of the split peaks with the gradual closing of the induced energy gap (mini-gap) in the F layer (Figure 4a). The influence of the perpendicular magnetic scattering can be observed in Figure 4c. While increasing the scattering rate α*_x_* tends to suppress the split peaks, perpendicular magnetic scattering also moves the peaks towards the Fermi energy destroying the mini-gap. This can be explained in terms of the additional effective exchange field in the system, which is due to non-zero perpendicular scattering rates [84]. Summarizing the results of the calculations, it is obvious that the magnetic scattering tends to destroy the proximity-induced superconductivity in the F layer. Such an effect becomes clear from a more detailed analysis of the linearized Usadel equation (Equation 2) in the low-proximity limit. In this case, the anomalous Green’s function is dependent on the exchange field *h* and the magnetic scattering rates, which apparently are pair breaking.

Figure 4b shows that a smaller spin–orbit scattering time leads to the vanishing of the peak splitting in the subgap region. In contrast, though the spin–orbit scattering destroys the double peak structure due to an exchange field smearing them into one peak, it does not produce a destructive effect on the mini-gap magnitude (see below Figure 6b). This feature has been reported previously [85].

#### Low interface transparency (γ*_B_* = 50)

**Figure 5:**
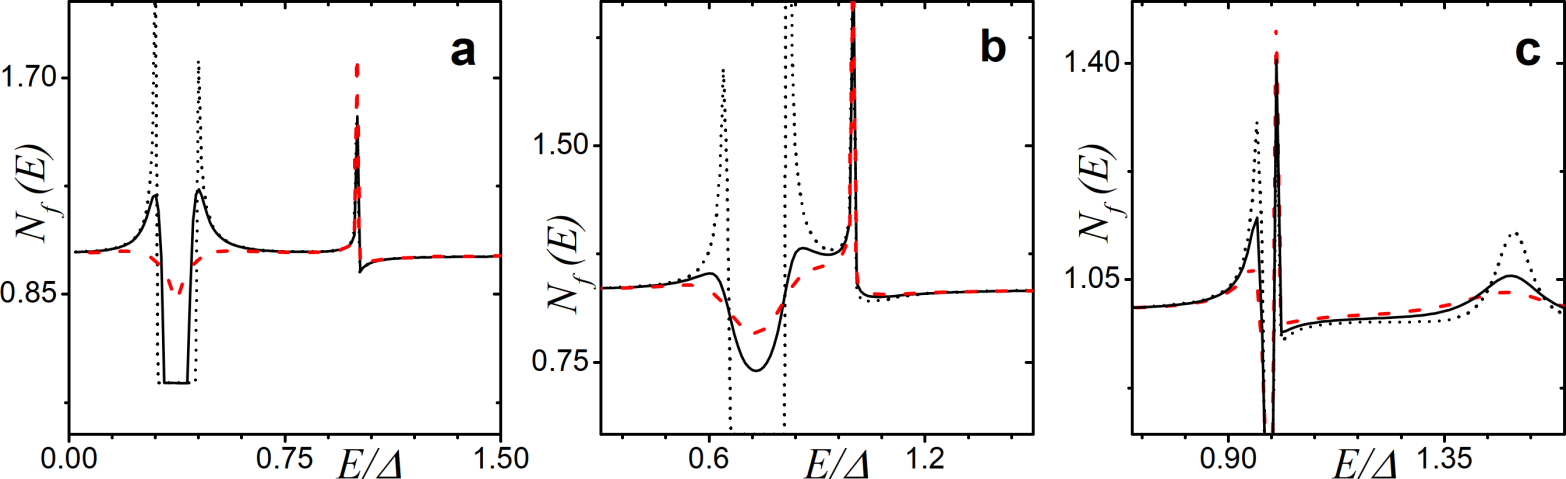
The DOS *N* *_f_*(*E*) at the free boundary of the F layer in the SF bilayer in the presence of magnetic scattering, calculated numerically for the low-transparency interface with γ*_B_* = 50. Plot (a) corresponds to α*_z_* ≠ 0 and *h* = 0.4Δ: α*_z_* = 0.01 (black solid line) and α*_z_* = 0.1 (red dashed line). Plot (b) corresponds to α*_so_* ≠ 0 and *h* = 0.8Δ: α*_so_* = 0.05 (black solid line) and α*_so_* = 0.1 (red dashed line). Plot (c) corresponds to α*_so_* ≠ 0 and *h* = 1.5Δ: α*_so_* = 0.05 (black solid line), α*_so_* = 0.1 (red dashed line). The black dotted line represents *N* *_f_*(*E*) in the absence of any scattering.

Now we focus on the limit of a highly resistive SF interface and investigate the effects of a spin-dependent scattering. As expected, the influence of the adjacent superconducting layer on the DOS is rather limited due to low transparency of the interface (Figure 5). It can be seen that the mini-gap is hardly recognizable in the cases of both subgap values of *h* (Figure 5a,b) and *h >* Δ (Figure 5c). From the plots, we can say that the finite scattering rates suppress the DOS features in the case of low interface transparency as well (Figure 5, solid and dashed lines). Even the DOS peak at *E* =*h* is suppressed substantially (Figure 5c). However, a closer examination shows that all the scattering rates slightly differ in the way they modify the DOS structure.

**Figure 6:**
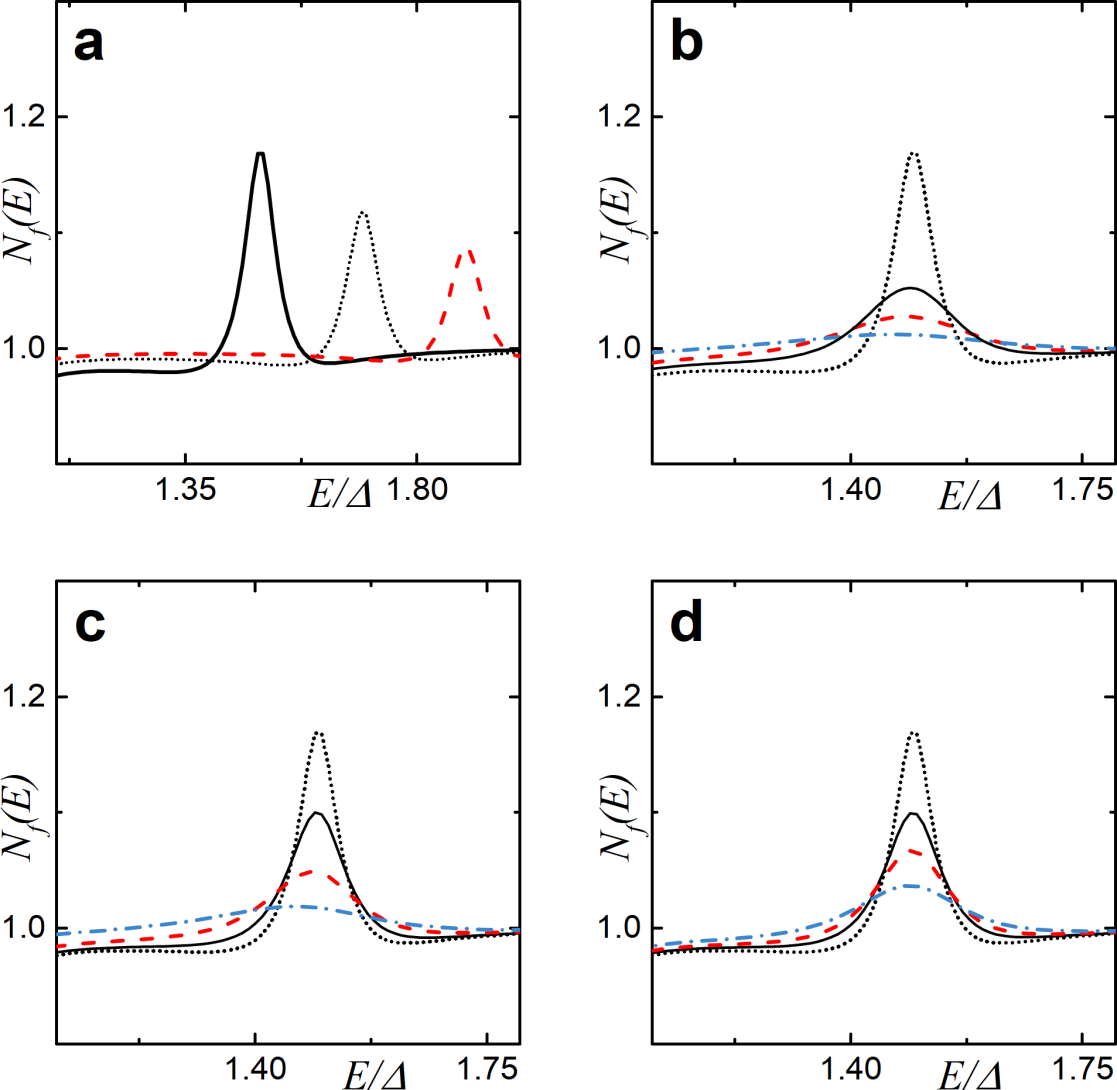
The peak at *E* = Δ in the DOS calculated numerically for three different *h* (a): *h* = 1.4Δ (black solid line), *h* = 1.6Δ (black dotted line), and *h* = 1.9Δ (red dashed line). The influence of different scatterings on the DOS peak at *E* =*h* in the SF structure. Here, γ*_B_* = 50, *d* *_f_* = 0.5ξ*_f_*, and the exchange field is *h* = 1.5Δ for plots (b)–(d). In plots (b)–(d), the black dotted line represents the DOS calculated in the absence of any scattering. Plot (b) corresponds to the case of nonzero α*_z_*: α*_z_* = 0.05 (black solid line), α*_z_* = 0.1 (red dashed line), and α*_z_* = 0.2 (blue dash-dotted line). Plot (c) corresponds to the case of nonzero α*_so_*: α*_so_* = 0.02 (black solid line), α*_so_* = 0.06 (red dashed line), and α*_so_* = 0.15 (blue dash-dotted line). Plot (d) corresponds to the case of nonzero α*_x_*: α*_x_* = 0.02 (black solid line), α*_x_* = 0.04 (red dashed line), and α*_x_* = 0.08 (blue dash-dotted line).

We would like to discuss the effect of scattering on the DOS peak located at the exchange energy in more detail. As we have mentioned above, one of the interesting features in the DOS of the considered system is the peak at *E* =*h* (Figure 6a). In Figure 6, we demonstrate the influence of different scattering rates on the DOS peak at *E* =*h* using the numerically obtained results. The remaining parameters used for calculations here are γ*_B_* = 50, *d* *_f_* = 0.5ξ*_f_*, and *h* = 1.5Δ. In Figure 6b, the plot for different values of α*_z_* is shown. It can be noticed that the uniaxial magnetic scattering not only suppresses the peak but also slightly shifts the DOS peak towards *E* = 0. The spin–orbit scattering has a similar effect on the peak, though α*_so_* has a stronger effect on the peak height compared to α*_z_* as it can be noticed from Figure 6c. In both cases above, the DOS peak also smears as any of the scattering rates increases. The effect of the perpendicular magnetic scattering α*_x_* is indicated in Figure 6c.

### Analytical result for the interfaces with low transparency and qualitative picture

Here, we employ the analytical expression (Equation 20) obtained in the limit of low proximity and thin F layer. Considering the problem in such limit makes it possible to use a simple expression for the qualitative description of the corresponding scattering effects on the DOS structure. It should not be forgotten that the linearized solution of the form in Equation 20 is quite limited in its application. In our case, it is valid when γ*_B_* ≫ 1 and *d* *_f_* ≪ min(ξ*_f_*,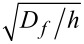 ), which is true for γ*_B_* = 50. This tunneling limit is experimentally feasible. Thus, our result could easily be applied.

**Figure 7:**
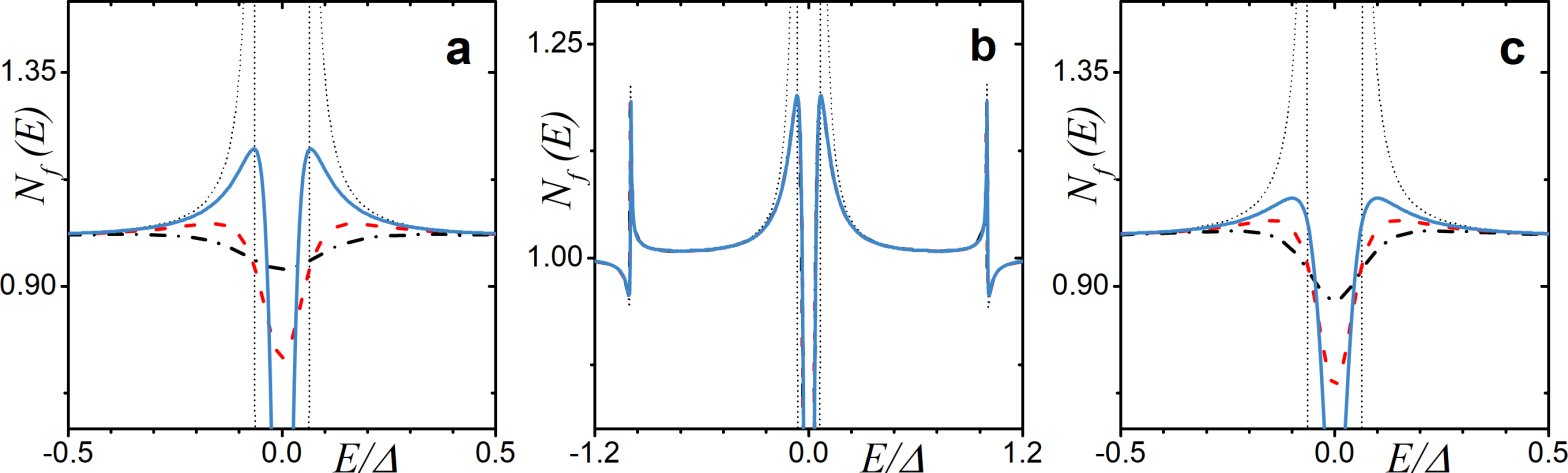
The DOS calculated analytically in the limit of low proximity and thin adjacent normal metal layer *h* = 0 with: (a) finite uniaxial magnetic scattering α*_z_* *>* 0 (α*_x_* = α*_so_* = 0); (b) spin–orbit scattering α*_so_* *>* 0 (α*_z_* = 0.02 and α*_x_* = 0); and (c) magnetic scattering α*_x_* *>* 0 (α*_z_* = 0.02 and α*_so_* = 0). The curves have been calculated for γ*_B_* = 50 and *d* *_f_* = 0.5ξ*_f_*. In the plots, the blue solid line corresponds to α*_i_* = 0.02, the red dashed line corresponds to α*_i_* = 0.05, and the black dash-dotted corresponds to α*_i_* = 0.1, where *i* is the corresponding scattering rate. The faint black dotted line corresponds to the analytical solution of Equation 17.

In Figure 7, the DOS calculated analytically via Equation 20 is illustrated. Here, we focus on the case of zero exchange field *h* = 0 to investigate the impact of each type of scattering on the mini-gap. We plot the analytically obtained DOS for the SN case in the absence of any scattering for comparison (black dotted line) as well. From the figure, one can see that the spin–orbit scattering does not affect the DOS in any way (Figure 7b). This effect has been shown before numerically in [85]. In contrast, both nonzero types magnetic of scattering, α*_x_* and α*_z_*, have a strong effect on the mini-gap, leading to its complete vanishing at some value of α (Figure 7a,c).

Making comparisons with previous results [84–85], we can say that there is a qualitative agreement in the DOS behavior. In the case of large DOS variations and, especially, singularities, the analytical model introduced above may fail. Nevertheless, we can explain major features of *N* *_f_*(*E*) in the presence of a spin-dependent scattering. Examining the linearized Usadel equations, we can analyze the anomalous Green’s functions by studying even-frequency spin-singlet (*f* *_s_* ∝ (θ*_f↑_* + θ*_f↓_*)/2) and odd-frequency spin-triplet (*f* *_t_* ∝ (θ*_f↑_* − θ*_f↓_*)/2) components. When there is an F layer with a relatively high rate of parallel magnetic scattering, we can simplify the linearized Usadel equation and obtain


[22] $$\frac{D_{f}}{2}\frac{\partial^{2}\theta_{f↑\left(↓\right)}}{\partial x^{2}}=\frac{1}{\tau_{z}}\theta_{f↑\left(↓\right)}.$$

From this equation, we can find that θ*_f↑_* = θ*_f↓_* = 0, leading to supression of both the singlet and triplet components. In the presence of large in-plane magnetic scattering α*_x_*, we obtain θ*_f↑_* = −θ*_f↓_*, which leads to *f* *_s_* = 0, whereas the triplet component *f* *_t_* is nonzero. This can be understood in a similar way from the linearized Usadel equation


[23] $$\frac{D_{f}}{2}\frac{\partial^{2}\theta_{f↑\left(↓\right)}}{\partial x^{2}}=\frac{1}{\tau_{x}}\left(\theta_{f↑}+\theta_{f↓}\right).$$

Since the energy gap is defined by the singlet correlations, we observe a detrimental effect of the in-plane scattering on the mini-gap magnitude (Figure 7c). In contrast, in the limit of strong spin–orbit scattering α*_so_*, the Usadel equation in F layer reads


[24] $$\frac{D_{f}}{2}\frac{\partial^{2}\theta_{f↑\left(↓\right)}}{\partial x^{2}}=\pm \frac{1}{\tau_{so}}\left(\theta_{f↑}-\theta_{f↓}\right),$$

which results in θ*_f↑_* = θ*_f↓_*, causing strong suppression of the triplet component and not the singlet one, which in turn explains the robustness of the mini-gap (Figure 7b). It can be demonstrated that the suppression of the triplet component due to spin–orbit scattering can actually lead to the appearance of the mini-gap at finite exchange fields *h*.

One of the possible ways of observing the DOS features is the examination of the current–voltage characteristics. Utilizing the Werthamer expression for the quasiparticle current in tunneling junctions, we can calculate the *I*–*V* curves for an SFIFS junction. The current then reads


[25] $$I=\frac{1}{eR}\int_{-\infty }^{\infty }\text{d}EN_{f1}\left(E-eV\right)N_{f2}\left(E\right)\left[f\left(E-eV\right)-f\left(E\right)\right].$$

**Figure 8:**
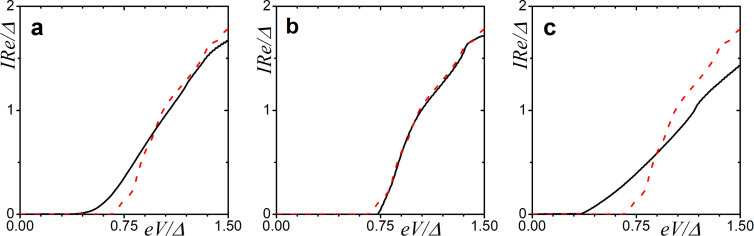
Current–voltage characteristics of a SFIFS junction in the presence of a spin-dependent scattering. The plots correspond to intermediate interface transparency γ*_B_* = 5, *h* = 0.1Δ, and *d* *_f_* = 0.5ξ*_f_*. Plot (a) corresponds to α*_z_* = 0.1, plot (b) corresponds to α*_so_* = 0.13, and plot (c) corresponds to α*_x_* = 0.2. The red dashed line represents the case of zero scattering.

Here, *N* *_f1,2_*(*E*) is the density of states (DOS) in the corresponding ferromagnetic layer at *x* = 0, *f*(*E*) = [1 + e*^E/T^*]^−1^ is the Fermi–Dirac distribution function, and *R* =*R* *_B_*_0_ is the resistance across the FIF interface. Both densities of states *N* *_f_*_1_ *_,_*_2_(*E*) are normalized to their values in the normal state. The abovementioned effects of spin-dependent scattering have a direct influence on the current. Figure 8 demonstrates the current–voltage characteristics of the SFIFS junction calculated in the presence of parallel magnetic (Figure 8a), spin–orbit (Figure 8b), and perpendicular magnetic scattering (Figure 8c). From the plots, we can notice that while a magnetic scattering destroys the mini-gap, the spin–orbit scattering slightly enhances it.

**Figure 9:**
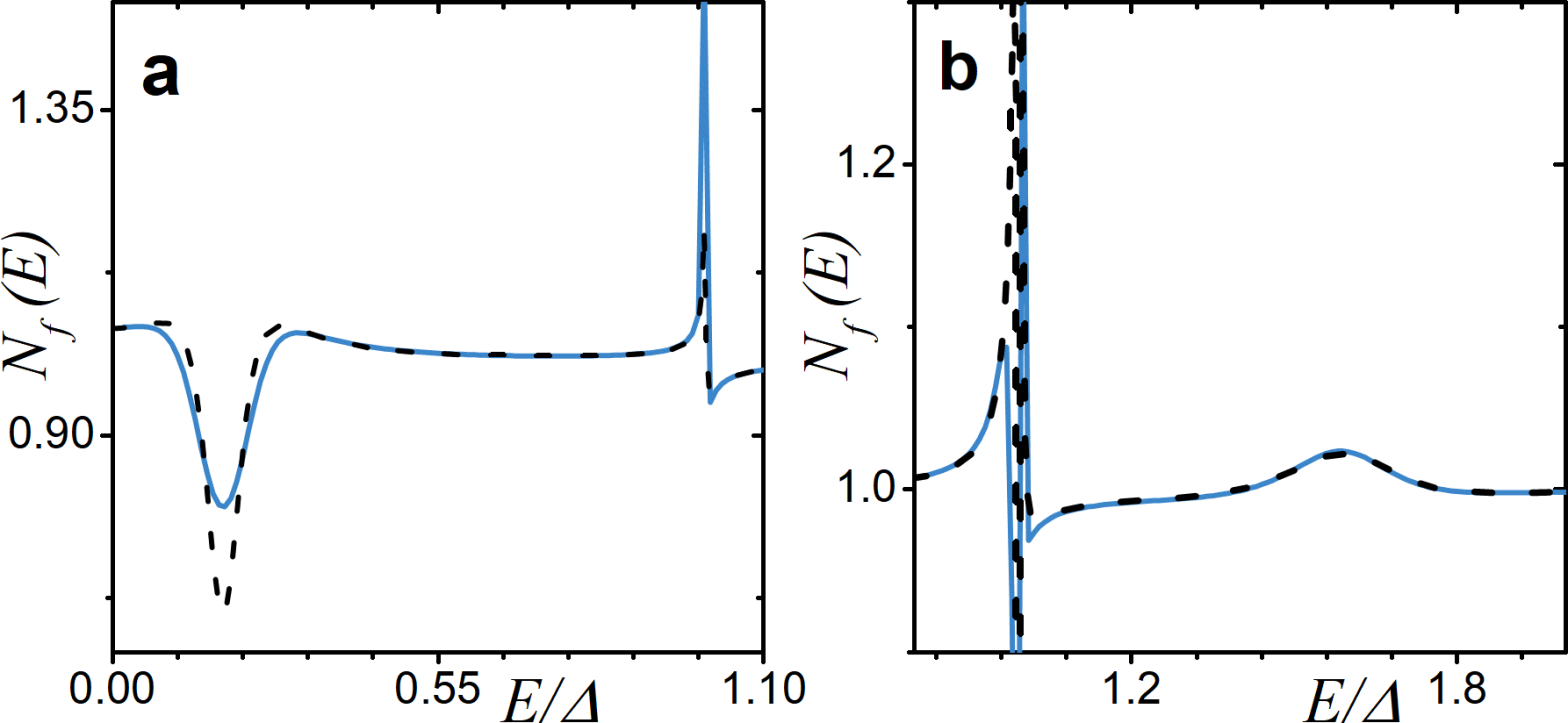
Comparison of the analytical result in the limit of low proximity and a thin adjacent ferromagnetic layer (black dashed line) with the DOS obtained numerically (blue solid line) for two different values of exchange field *h*. The parameters are γ*_B_* = 50 and *d* *_f_* = 0.5ξ*_f_*. Plot (a): *h* = 0.2Δ and α*_z_* = α*_x_* = α*_so_* = 0.02. Plot (b): *h* = 1.6Δ and α*_z_* = 0.02 and α*_so_* = α*_x_* = 0.04.

Finally, we compare the analytically derived and numerically calculated DOS in the case of an SF junction with a thin F layer and low-transparency interface. The corresponding result is shown in Figure 9. We can observe a fairly good agreement between the numerical and the analytical calculations. As expected, the analytical expression in Equation 20 cannot describe the features of *N* *_f_*(*E*), which are relatively large in scale compared to unity.

## Conclusion

We have formulated a model that takes into account magnetic and spin–orbit scattering processes in the framework of the quasiclassical Green’s function approach in the diffusive limit. Based on these equations, the local density of states has been calculated numerically. Applying the developed numerical solution, we have studied some previously overlooked features such as the influence of the scattering rates on the peak at *E* =*h*. Moreover, we provide a relatively simple expression to calculate the DOS analytically in the presence of magnetic scattering α*_z_* for thin F layers. In addition, the analytic solution for the anomalous Green’s function has been derived in the limit of low proximity and a thin ferromagnetic layer. Based on this solution, we have been able to present analytical results for the DOS taking into account all spin-dependent scattering. We have demonstrated that the analytical result is in qualitative agreement with the numerical predictions, including previously published findings.
